# CircRNA-5692 inhibits the progression of hepatocellular carcinoma by sponging miR-328-5p to enhance DAB2IP expression

**DOI:** 10.1038/s41419-019-2089-9

**Published:** 2019-11-27

**Authors:** Zhenguo Liu, Yaqun Yu, Zebing Huang, Yi Kong, Xingwang Hu, Wei Xiao, Jun Quan, Xuegong Fan

**Affiliations:** 1 Department of Infectious Disease, Hunan Key Laboratory of Viral Hepatitis, Xiangya Hospital, Central South University, Changsha, 410008 China; 2 Department of Infectious Disease, the Third Xiangya Hospital, Central South University, Changsha, 410013 China; 3 Department of Hepatobiliary and Pancreatic Surgery, the Affiliated Hospital of Guilin Medical College, Guilin, 541002 China; 4 The Department of Hepatopancreatobiliary Medicine, Hunan Cancer Hospital, Changsha, 410013 China

**Keywords:** Cancer therapy, Liver cancer

## Abstract

Circular RNAs (circRNAs), one kind of noncoding RNAs, can interact with miRNA and transcription factors to regulate gene expression. However, little is known on which circRNA is crucial for the pathogenesis of hepatocellular carcinoma (HCC). CircRNA expression profile was analyzed by a microarray. Regulatory gene targets were predicted by bioinformatics analysis and validated by luciferase assay. Their expression was determined by qRT-PCR and Western blotting. DNA methylation was determined by methylation-specific PCR. Gene knockdown and overexpression were mediated by lentivirus-mediated shRNA and transfection with plasmids for cDNA expression, respectively. MTT assay, wound-healing assay, transwell invasion assay, and flow cytometry were used to determine malignant behaviors of HCC cells. HCC xenograft mouse model was used to determine the in vivo effects of circRNA-5692. CircRNA-5692 expression was downregulated in HCC tissues, and circRNA-5692 overexpression attenuated the malignant behaviors of HCC cells. Bioinformatics predicted that circRNA-5692 interacted with miR-328-5p, which targeted the *DAB2IP* mRNA. Actually, miR-328-5p promoted the malignant behaviors of HCC cells, while DAB2IP had opposite effects. Moreover, circRNA-5692 overexpression inhibited the growth of xenograft HCC tumors in vivo by decreasing miR-328-5p expression to enhance DAB2IP expression. In conclusion, the circRNA-5692–miR-328-5p–DAB2IP regulatory pathway inhibits the progression of HCC. Our findings may provide potential new targets for the diagnosis and therapy of HCC.

## Introduction

Hepatocellular carcinoma (HCC) accounts for 90% of primary liver carcinomas in the world^1,2^. HCC is one of the leading causes of malignancy in humans, and has high morbidity and mortality rates^3^. Despite of significant advance in therapeutic strategies for HCC, the 5-year survival rate of HCC patients remains low^4^. More importantly, little is known on the molecular pathogenesis and therapeutic targets of HCC. Therefore, understanding the pathogenic process of HCC and its regulatory mechanisms will be of great significance in management of HCC.

Circular RNAs (circRNAs), microRNAs (miRNAs), and other noncoding RNAs can regulate cell activities^5^. CircRNAs are covalently closed continuous loop type of single-stranded RNAs in mammalian cells ubiquitously and regulate gene expression^6^. Previous studies have shown that circRNAs can regulate malignant behaviors, including the proliferation, migration, invasion, and apoptosis of cancer cells^7–10^. For example, circRNA hsa_circ_0000096 regulates the proliferation and migration of gastric cancer cells by modulating the gene expression of cyclin D1, cyclin-dependent kinase-6 (CDK-6), matrix metalloproteinase-2 (MMP-2), MMP-9, and E-cadherin^8^. The circRNA PVT1 and hsa_circ_0067934 act as oncogenic factors to promote the proliferation of gastric cancer and esophageal carcinoma cells^9,10^. CircRNAs can act as competing endogenous RNAs (ceRNAs) to sponge their target miRNAs via direct binding to miRNAs, and modulate their activity, regulating the miRNA-targeted gene expression^11,12^. A recent study has indicated that circRNA CiRS-7 can promote the growth and metastasis of esophageal squamous cell carcinoma by sponging miR-7 and suppressing its activity^13^. The circRNA Vav3 can sponge gga-miR-375 to affect the YAP1 expression, by promoting the epithelial mesenchymal transition (EMT) process^14^. Furthermore, circRNAs can interact with transcription factors and other proteins to form circRNPs, and modulate their function^15–17^. A recent study suggests that circRNAs are important for the initiation, migration, and invasion of HCC^18^. However, which circRNA and how it regulates the malignant behaviors of HCC have not been clarified.

In this study, we screened differentially expressed circRNAs that were significantly downregulated in HCC tissues and identified that circRNA-5692 overexpression effectively attenuated the malignant behaviors of HCC. Furthermore, we explored its potential targeted miRNAs and found that circRNA-5692 acted as a ceRNA to sponge oncogenic miR-328-5p to enhance tumor suppressor DAB2IP (Ras (MIM 190020) GTPase-activating protein) expression, attenuating the malignant behaviors of HCC in vitro and in vivo.

## Materials and methods

### HCC specimens and microarray of circRNAs

The experimental protocols were approved by Ethics Committee of Xiangya Hospital of Central South University (201502012). We collected 92 pairs of surgical or biopsied HCC and adjacent non-tumor specimens in Xiangya Hospital of Central South University, Hunan Cancer Hospital, and Affiliated Hospital of Guilin Medical College from February 2017 to June 2018. All samples were collected with patient consent and signed informed consent. The HCC patients were diagnosed, based on the practice guidelines of the American Association for the Study of Liver Diseases (AASLD). Their liver specimens were evaluated by pathologists and their clinical stages were determined, according to the BCLC classification. HCC patients with the following conditions were excluded: (1) patients ≤ 18 or ≥ 70 years of age or without full civil capacity; (2) patients with a history of preoperative anticancer radiotherapy or chemotherapy, biological, immune, and traditional Chinese medicine; (3) patients with incomplete postoperative follow-up data; (4) patients with a history of another organ malignancy, or systemic immune disease. Their tissue RNA was extracted by using a RNeasy Mini Kit (Qiagen, Hilden, Germany), and the RNA concentrations were measured by a NanoDrop 1000 spectrophotometer (ThermoScientific, Waltham, MA). The contained circRNAs were enriched and digested with RNase A and reversely transcribed into cRNA by using fluorescent reagents and random primers. The circRNA profile was determined by Microarray by using Human circRNA Arrays (8 × 15 K, Arraystar, Rockville, MD, USA). A change of ≥ 2-fold in the circRNA level was defined as differential expression. The data and potential interaction with miRNAs were analyzed by using R software limma and Arraystar program (Arraystar).

### Cell culture

Human HCC HCCLM3, Huh-7, HepG2, cervical cancer Chang Liver cells, non-tumor human embryotic kidney HEK293T, liver WR168, and LX-2 cells were obtained from American Type Culture Collection (ATCC, Manassas, VA, USA). The cells were cultured in DMEM containing 20% fetal bovine serum (FBS), 2 mM l-glutamine, 100 units/ml of penicillin, and 100 µg/ml of streptomycin (complete medium, Gibco, Aukland, New Zealand) at 37 °C in an incubator of 5% CO_2_.

### Transfection and transduction of cells

The plasmid pHBLV-CMV-Cicr-MCS-EF1-circRNA-5692, its derived lentivirus, miRNA-328-5p mimics, miRNA-328-shRNA, miR-1207-5p mimic, and their negative control miRNAs, their relevant miRNA-derived lentiviruses, plasmids for expression of DAB2IP, DAB2IP-specific shRNA, and their relevant lentiviruses were obtained from Shanghai Hanheng Biotech (Shanghai, China). HCC cells were transduced with individual types of lentivirus at a multiplicity of infection (MOI) of 10 in the presence of 5 µg/ml puromycin or transfected with the specific plasmid by using Lipofectamine 2000 (ThermoFisher).

### Quantitative PCR and methylation-specific PCR

The different groups of cells were harvested and their total RNAs were extracted by using Trizol reagent (Invitrogen). The RNA samples (1–3 µg each) were reversely transcribed into cDNA by using the Superscrpt II kit (Invitrogen). The relative levels of target gene RNA transcripts were determined by qRT-PCR by using the SYBR Green mix (Kakara, Dalian, China) and specific primers in an ABI 7500 thermocycler (Applied Biosystems). The sequences of primers were forward 5′-GCCTGAATGATGACTGCTGA-3′ and reverse 5′-GGTAACAGAAGCGCCTGAAG-3′ for hcirc-0001727-220; Forward 5′-GCTCGACCTGAAGCTGAGTA-3′ and Reverse 5′- CTTGGAGTTCAGCAGGAAGC-3′ for hcirc-0005692-172; Forward 5′-CATTGCCCCATGTGAAGTC-3′ and Reverse 5′-GGTGCCCCTGGAGATTTTAG-3′ for hcirc-0028861-109; Forward 5′-GTATGGTGTGGCTTGTGTGG-3′ and Reverse 5′- GCTGCAATCCTCAGAGAAGG-3′ for hcirc-0034762-169; Forward 5′-GAAATGCCCCTTCACTGGTA-3′ and Reverse 5′-TGTGACGATGTCACCGATCT-3′ for hcirc-0051908-208; Forward 5′-TTCTCCCACTCTGGGCTCT-3′ and Reverse 5′-GAGTCTTGGGTCTCCCAGAA-3′ for hcirc-0092283-211; Forward 5′-CCAGGTGGTCTCCTCTGA-3′ and Reverse 5′-GCCAAATCGTTGT-3′ for GAPDH; Forward 5′-AGTGGCAGGGAGGCTGG-3′ and Reverse 5′- GTCGTATCCAGTGCAGGGTCCGAGGTATTCGCACTGGATACGACCCCCTC-5′ for hsa-miR-1207-5p; Forward 5′- GGGGGCAGGAGGGGC-3′ and Reverse 5′-GTCGTATCCAGTGCAGGGTCCGAGGTATTCGCACTGGATACGACCCCTGA-3′ for hsa-miR-328-5; Forward 5′-GGACAGCAGGCACAGACA-3′ and Reverse 5′- GTCGTATCCAGTGCGTGTCGTGGAGTCGGCAATTGCACTGGATACGACACTGCC-3′ hsa-miR-214-3p; Forward 5′-TGAGGGGCTGGCTTTCC-3′ and Reverse 5′-GTCGTATCCAGTGCGTGTCGTGGAGTCGGCAATTGCACTGGATACGACGACCAG-3′ for has-miR-185-3P; Forward 5′-CTCGCTTCGGCAGCACA-3′ and Reverse 5′-AACGCTTCACGAATTTGCGT-3′U6; Forward 5′- GGTGGGGACAAGACAGAAGA-3′ and Reverse 5′-CTAAAAGCCCCTTCCCAGAG-3′ for DAB2IP; Forward 5′- CCTGTCTCAGGTGTGAGCAA-3′ and Reverse 5′-GGACTGACCCCACACTCTGT-3′ for nuclear factor I C (NFIC); Forward 5′-TGGAAGGTCAGGGAAACATC-3′ and Reverse 5′-GCTGACTGTGAACTCCCTCC-3′ for IL-27; Forward 5′- CTAAAGAGACCGCGTTCCAG-3′ and Reverse 5′-TGGTGACTGAGGAAGGAAGG-3′ for hypermethylated in cancer 1 (HIC1). For the methylation-specific PCR, the DAB2IP promoter region was amplified by using primers of M-Forward 5′-TTTTTTAATGTTTTTAGTTAGGTTGC-3′ and M-Reverse 5′-CTCCTTTTATATTCCATCTAACGAC-3′.

### Luciferase assay

Dual luciferase reporter system psiCHECK^TM^ (Fisher Scientific) was used for luciferase assay. The circRNA-5692 (WT) and its mutant sequences were cloned into the plasmid psiCHECK2. HEK293T cells (4 × 10^4^ cells/well) were cultured in 24-well plates overnight and transfected with 400 ng of psiCHECK vector, psiCHECK-circRNA-5692WT, psiCHECK-circRNA-5692 mutant, psiCHECK-DAB2IP WT, or psiCHECK-DAB2IP mutant, together with the plasmid for Renilla luciferase expression by lipofectamine 2000. One day later, the cells were lysed and their luciferase activities were measured by using Dual-luciferase reporter assay system (Promega). Some luciferase assays were performed after co-transfection with miR-328-5P mimics or its mutant.

### Western blotting

The different groups of cells were lysed in lysis buffer supplemented with protease inhibitors (Roche, Indianapolis, USA) and centrifuged. The protein concentrations were determined by a BCA kit (Pierce, Rockford, USA). The cell lysates (10–20 µg/lane) were separated by sodium dodecyl sulfate polyacrylamide gel electrophoresis (SDS-PAGE) on 10–12% gels and transferred to polyvinylidene difluoride (PVDF) membranes (Millipore, Billerica, USA). After blocking with 5% bovine serum albumin (BSA), the membranes were probed with primary antibodies for 4 h at 37 °C and detected with horseradish peroxidase (HRP)-conjugated secondary antibodies (1:5000, AP308P, Sigma-Aldrich), followed by visualization by using enhanced chemiluminescent reagents. The primary antibodies included anti-E-cadherin (1:1000, ab1416, Abcam), anti-Vimentin (1:1000, ab137321, Abcam), anti-β-actin (1:1000, ab227387, Abcam), and anti-Snail (1:1000, ab53519, Abcam). The relative levels of each protein expression were determined by densitometric analysis by using ImageJ software.

### MTT assay

The proliferation of HCC cells was measured in sextuplicate by MTT assay by using Cell Proliferation Kit (Sigma-Aldrich) according to the manufacturer's instructions.

### Flow cytometry

The frequency of apoptotic HCC cells was examined by flow cytometry by using FITC-labeled Annexin V and propidium iodide (PI) in a flow cytometer (Attune NxT, ThermoFisher).

### Wound-healing assay

The wound healing of HCC cells was measured. HCC cells were cultured in six-well plates up to ~100% confluence. The cells were starved for 6 h and wounded with a sterile 200-μL pipette tip. After being washed, the cells were cultured for 12 h and photoimaged before and after 12-h culture.

### Transwell invasion assay

The invasion of different groups of HCC cells was determined by transwell invasion assay. Briefly, HCC cells (10^5^ cells/well) were cultured in serum-free medium in the top chamber that had been loaded with Matrigel. The bottom chambers were filled with 600 μL of complete medium. After 24–48 h of incubation, the cells on the upper surface of the top chamber were removed with a cotton swab, and the invaded cells on the bottom surface of the top chamber were stained with Harris hematoxylin solution (Sigma) and photoimaged under a light microscope.

### Bioinformatics analysis

The potential target miRNAs of circRNA-5692 were predicted by using the tool in bioinformatics database CircNet and further predicted by Shanghai Kangcheng Biotech. The potential target genes of miR-328-5p were predicted by using the miRDB (http://mirdb.org/) and TargetScanHuman (www.targetscan.org).

### HCC animal model

C57BL/6 nude mice were obtained from Hunan Slack Jingda Experimental Animal (Changsha, China, the experimental animal production license number: SCXK (Xiang) 2016-0002) and injected with 4 × 10^6^ WT Huh-7 cells (control), the vehicle-transfected Huh-7 cells, or circRNA-5692-overexpressed Huh-7 cells (15 nude mice were randomly divided into 3 groups, 5 in each group). The growth of implanted HCC tumors was monitored for their volumes every 5 days up to 30 days post inoculation. The mice were killed and their xenografts were photoimaged and measured.

### Statistical analysis

Data are expressed as mean ± standard error of the mean (SEM). Comparisons among groups were analyzed by one-way ANOVA and post hoc Tukey tests, and the differences between groups were analyzed by two-tailed t tests by using SPSS 10.0 for Windows. A *P*-value of < 0.05 was considered statistically significant.

## Results

### The expression of circRNA-5692 is downregulated in HCC

**Fig. 1:**
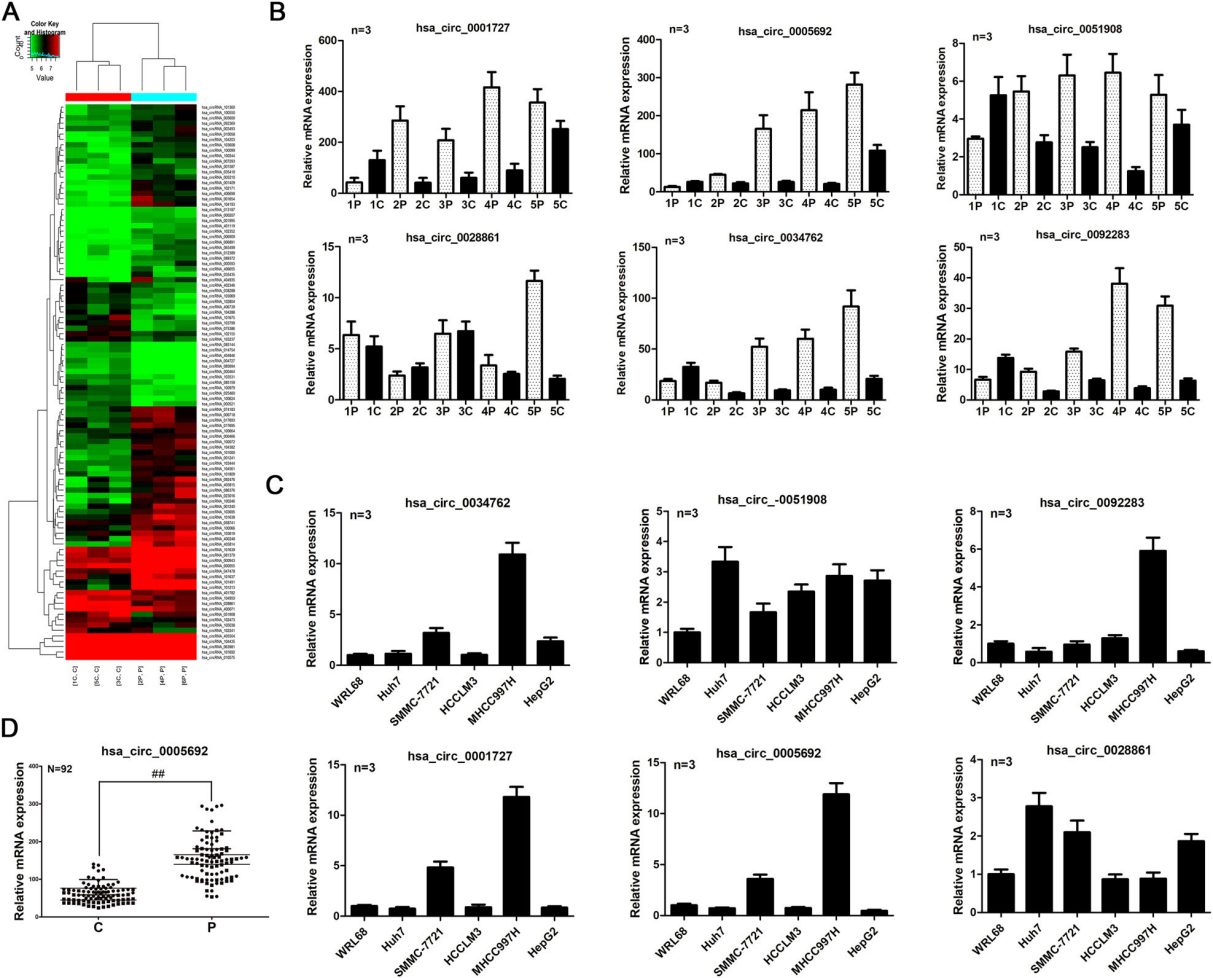
The differential expression of circRNAs in HCC tissues. The differential expression of circRNAs in three pairs of HCC and non-tumor liver tissues was analyzed by microarray. **a** The heat mapping of some differentially expressed circRNAs. **b** Quantitative RT-PCR analysis of relative expression levels of the indicated circRNAs in five pairs of specimens. **c** Quantitative RT-PCR analysis of relative levels of the indicated circRNAs in different types of cells. **d** Quantitative RT-PCR analysis of relative levels of has_circ_0005692 (circRNA-5692) in 92 pairs of HCC and non-tumor liver tissues. Data are a representative image or expressed as the mean or mean ± SEM of each group from three separate experiments. **P < 0.01. c cancer tissues, p para-non-tumor tissues.

To investigate the potential role of circRNAs in regulating the progression of HCC, 92 pairs of surgical and biopsied HCC and paracarcinoma non-tumor tissues were obtained, and the demographic and clinical charecteristics of those patients are shown in Table 1. The expression profile of circRNAs in HCC and non-tumor liver tissues was analyzed by microarray. In comparison with the non-tumor tissues, there were 103 differentially expressed circRNAs, including 32 upregulated and 71 downregulated in HCC tissues (Fig. 1a). Further analysis of four upregulated and four downregulated circRNAs validated six out of eight differential expression in five pairs of specimens in Fig. 1b, and fiveout of six circRNAs were differentially expressed in HCC Huh-7, SMMC-7721, HCCLM3, MHCC997H, and HepG2 cells (Fig. 1c). It was notable that the circRNA-5692 was encoded by the *GLIS2* gene and most significantly downregulated in five HCC tissues and HCC cells (Fig. 1b, c). Further analyses revealed that the relative levels of circRNA-5692 expression in 92 HCC tissues were significantly lower than those in the para-non-tumor liver tissues (*P* < 0.01, Fig. 1d). Stratification analyses indicated that the lower circRNA-5692 expression was significantly associated with abnormal higher levels of AFP (*P* = 0.001), cirrhosis history (P = 0.001), larger tumor size (*P* = 0.042), and distant metastasis (*P* = 0.025), but not other measures tested in this population. Hence, downregulated circRNA-5692 expression may be associated with the progression of HCC.

**Table 1:** Association between hsa_circ_0005692 expression and clinical parameters in HCC.

Items		Case No	Mean ± SD	*P*-value
Gender	Male	76	60.617 ± 24.631	0.102
	Female	16	71.906 ± 26.029	
Age	≤50	35	62.112 ± 21.268	0.889
	>50	57	62.868 ± 27.373	
AFP	<20 ng/ml	25	78.869 ± 29.081	0.001
	≥20 ng/ml	67	56.502 ± 20.577	
HBsAg	Positive	92		
	Negative	0		
Cirrhosis history	Positive	71	58.131 ± 21.492	0.001
	Negative	21	77.624 ± 30.709	
Tumor size	≤5 cm	55	66.939 ± 24.650	0.042
	>5 cm	37	56.101 ± 24.688	
Tumor number	1	70	65.287 ± 26.671	0.065
	≥2	22	53.969 ± 17.079	
Differentiation	I/II	37	68.733 ± 30.144	0.053
	III/IV	55	58.442 ± 20.320	
Distant metastasis	No	74	65.456 ± 25.831	0.025
	Yes	18	50.759 ± 18.013

### CircRNA-5692 overexpression suppresses the malignant behaviors of HCC cells

**Fig. 2:**
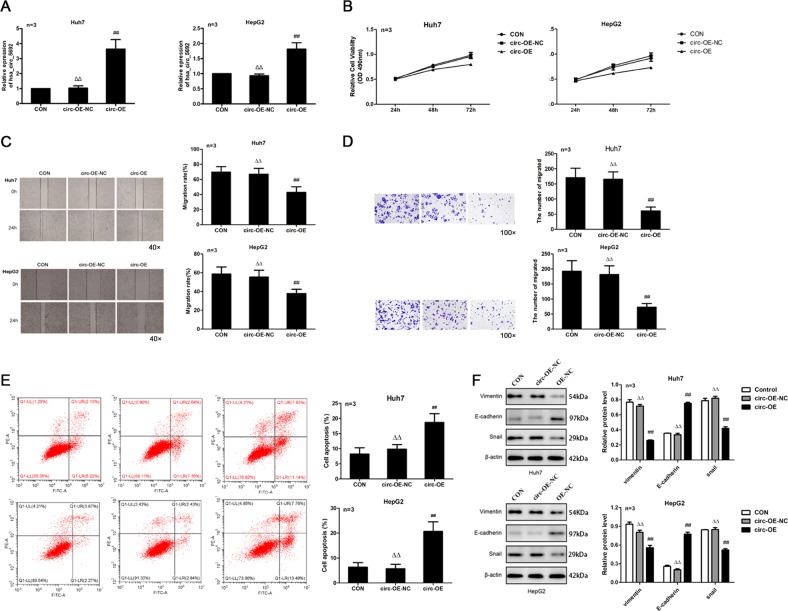
Induction of circRNA-5692 overexpression inhibits the proliferation, wound healing, and invasion of Huh-7 and HepG2 cells in vitro. Huh-7 and HepG2 cells were transduced with control lentivirus or virus for expression of circRNA-5692. The proliferation, wound healing, invasion, and apoptosis of HCC cells were measured. The relative levels of EMT-relevant protein expression were determined. **a** The relative levels of circRNA-5692 expression in HCC cells. **b** circRNA-5692 overexpression suppressed cell proliferation of Huh-7 and HepG2. **c** circRNA-5692 overexpression inhibited the wound healing of HCC cells. **d** circRNA-5692 overexpression attenuated the invasion of HCC cells. **e** circRNA-5692 overexpression promoted the apoptosis of HCC cells. **f** Western blotting analysis of the relative levels of the indicated protein expression in different groups of cells. Data are representative images, charts, or expressed as the mean ± SEM of each group from three separate experiments. ^##^*P* < 0.01 vs. CON; ^▵▵^*P* < 0.01 vs. circ-OE. OE overexpression, NC transduced with control lentivirus.

To investigate the potential functions of circRNA-5692 in regulating the progression of HCC, we generated circRNA-5692 stably overexpressing Huh-7 and HepG2 cells by transducing them with lenti-pHBLV-CMV-circRNA-5692 (Fig. 2a). In comparison with the controls, circRNA-5692 overexpression decreased the proliferation, wound healing, and invasion of both Huh-7 and HepG2 cells (*P* < 0.05, *P* < 0.01, Fig. 2b–d). Flow cytometry analysis indicated that circRNA-5692 overexpression increased the frequency of spontaneously apoptotic Huh-7 and HepG2 cells (*P* < 0.01, Fig. 2e). Western blot displayed that circRNA-5692 overexpression significantly increased the relative levels of E-cadherin expression, but decreased Vimentin and Snail expression in both Huh-7 and HepG2 cells. Thus, circRNA-5692 overexpression inhibited the malignant behaviors by attenuating the EMT process in HCC cells (Fig. 2f).

### CircRNA-5692 acts as a ceRNA to sponge miRNA-328-5p

**Fig. 3:**
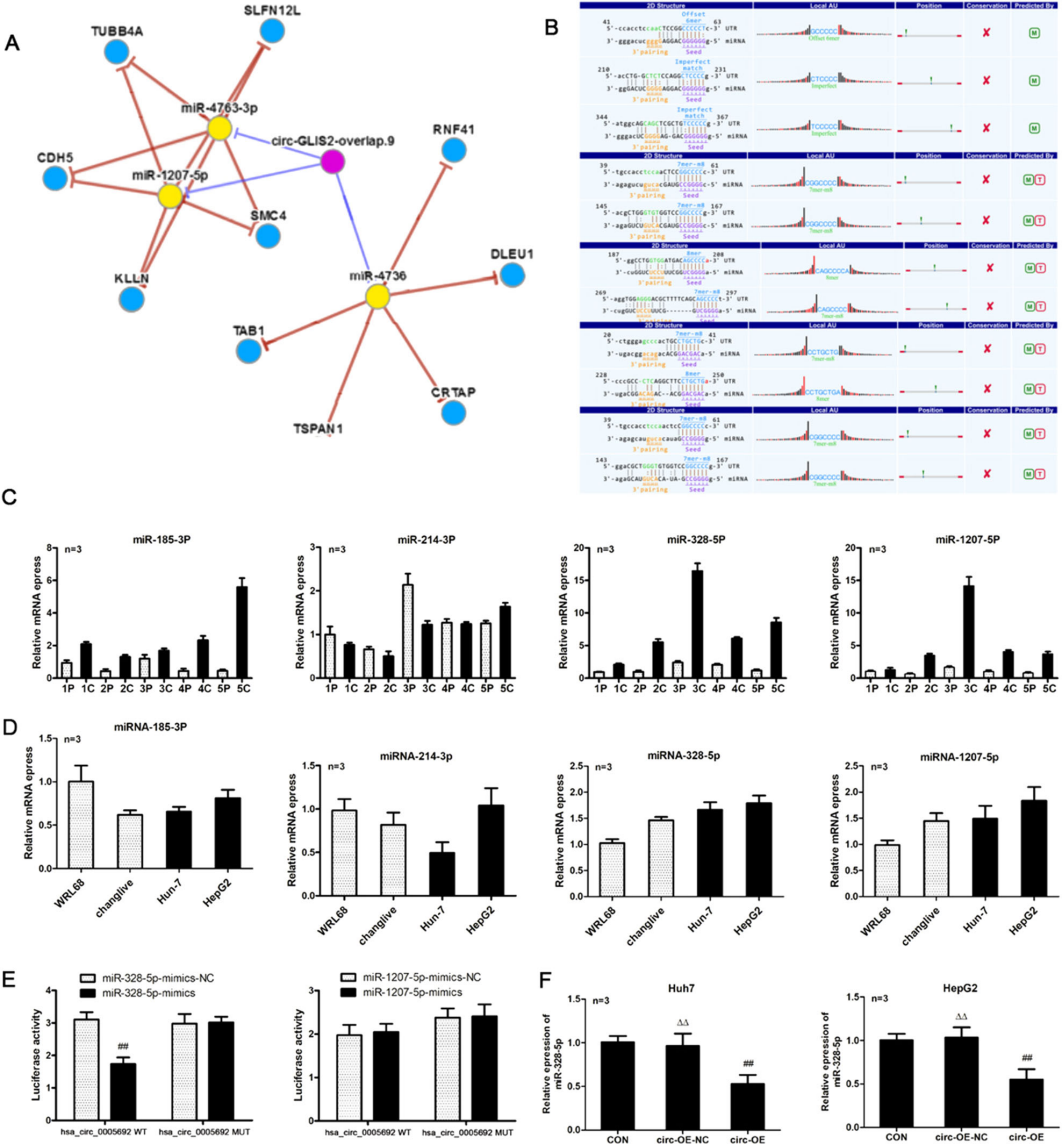
Bioinformatic analysis and validation of potentially targeted miRNAs of circRNA-5692 in HCC cells. **a** The potentially targeted miRNAs of circRNA-5692 were predicted by bioinformatics. **b** The potential binding sequences of circRNA-5692 to these miRNAs were analyzed by bioinformatics. **c** Quantitative RT-PCR analysis of the relative RNA levels of the indicated miRNAs in five pairs of HCC tissues. **d** Quantitative RT-PCR analysis of the relative RNA levels of the indicated miRNAs in the indicated cells. **e** miR-328-5p overexpression suppressed the circRNA-5692-regulated luciferase activity in HEK293T cells. **f** circRNA-5692 overexpression decreased miR-328-5p expression in HCC cells. Data are images, or expressed as the mean ± SEM of each group from three separate experiments. ^##^*P* < 0.01 vs. CON; ^▵▵^*P* < 0.01 vs. circ-OE. OE overexpression, NC transduced with control lentivirus. c cancer tissues, p para-non-tumor tissues.

It is well known that a circRNA can act as a ceRNA to sponge miRNAs to mitigate their inhibitory effect on the targeted mRNA expression^19^. To understand the action of circRNA-5692, we first predicted the potential targeting miRNAs in CircNet database by bioinformatics. As shown in Fig. 3a, circRNA-5692 (purple) was predicted to interact with miR-1207-5p, miR-4763-3p, and miR-4736 (yellow), which would target several gene mRNAs (blue) as well as miR-328-5p, miR-128-1-5p, miR-185-3p, miR-214-3p, and miR-128-2-5p (Fig. 3b). Given that miR-1207-5p, miR-328-5p, miR-185-3p, and miR-214-3p can regulate tumorigenesis^20–23^, they were selected for the potential miRNAs targeted by circRNA-5692. Actually, the expression of hsa-miR-1207-5p, hsa-miR-328-5p, and hsa-miR-185-3p increased in five HCC tissues, compared with their para-cancerous tissues (Fig. 3c). However, the expression of hsa-miR-214-3p decreased in four out of five HCC specimens. Similarly, the expression of miRNA-1207-5p and miRNA-328-5p, but not miR-185-3p and miR-214-3p, was upregulated in HCC Huh-7 and HepG2 cells, compared with Chang liver and non-tumor WR168 cells (Fig. 3d). Hence, miRNA-1207-5p and miRNA-328-5p were considered as the miRNA targets of circRNA-5692. Furthermore, luciferase assays indicated that co-transfection with miRNA-328-5p mimics, but not miRNA-328-5p mutant, or miRNA-1207-5p mimics, significantly mitigated the circRNA-5692-regulated luciferase activity in HEK293T cells (*P* < 0.01, Fig. 3e). Moreover, induction of circRNA-5692 overexpression significantly decreased the relative levels of miRNA-328-5p in both Huh-7 and HepG2 cells (*P* < 0.01, Fig. 3f). Collectively, these results suggest that circRNA-5692 may sponge miRNA-328-5p in HCC cells.

### MiRNA-328-5p enhances the malignant behaviors of HCC cells

**Fig. 4:**
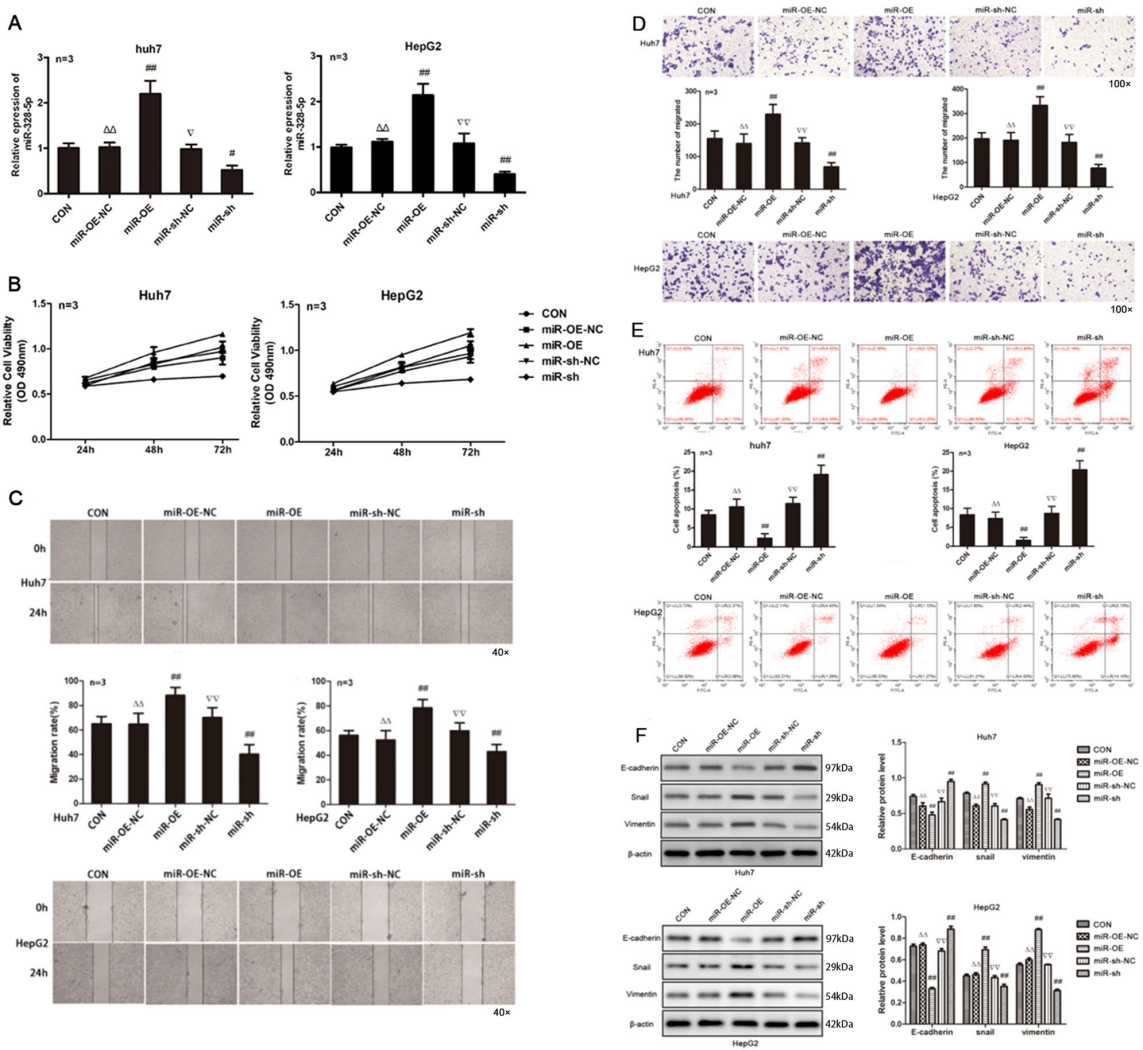
Altered miR-328-5p expression modulates the prolfieration, wound healing, invasion, and apoptosis of HCC cells. Huh-7 and HepG2 cells were transfected with control or miR-328-5p mimics or transduced with control lentivirus or lentivirus for expression of miR-328-5p-specific shRNA. The relative levels of miR-328-5p expression in HCC cells were determined with qRT-PCR, and the proliferation and wound healing were examined. **a** MiR-328-5p expression. **b** The prolfieration of HCC cells. **c** The wound healing. **d** The invasion of HCC cells. **e** The apoptosis of HCC cells. **f** Western blot analysis for the relative levels of EMT-relevant protein expression in HCC cells. Data are images, charts, or expressed as the mean ± SEM of each group from three separate experiments. ^##^*P* < 0.01 vs. CON; ^▵▵^*P* < 0.01 vs. miR-OE; ^▿▿^*P* < 0.01 vs. miR-sh. OE overexpression, NC transfected with control RNA or lentivirus, Sh transduced with lentivirus for miR-328-5p-specific shRNA.

To investigate the role of miRNA-328-5p in regulating HCC progression, we generated miRNA-328-5p stably overexpressing or silencing Huh-7 and HepG2 cells (Fig. 4a). Compared with the controls, miRNA-328-5p overexpression significantly enhanced the proliferation and wound healing of Huh-7 and HepG2 cells, while miRNA-328-5p silencing attenuated the proliferation and wound healing of Huh-7 and HepG2 cells in vitro (*P* < 0.01 for all, Fig. 4b, c). Similar patterns of invasion were detected in the different groups of cells (*P* < 0.01 for all, Fig. 4d). While miRNA-328-5p overexpression significantly decreased the frequency of apoptotic HCC cells, miRNA-328-5p silencing significantly increased it in both Huh-7 and HepG2 cells (*P* < 0.01 for all, Fig. 4e). Given that the EMT process is associated with cancer cell invasion, we tested the impact of altered miRNA-328-5p expression on the relative levels of E-cadherin, Snail, and Vimentin expression in HCC cells by Western blot (Fig. 4f). The results revealed that miRNA-328-5p overexpression significantly decreased the levels of E-cadherin, but increased Snail and Vimentin expression, while miRNA-328-5p silencing had opposite effects in Huh-7 and HepG2 cells. Such data indicated that miRNA-328-5p enhanced the malignant behaviors of HCC cells by promoting the EMT process.

### MiRNA-328-5p targets the DAB2IP expression

**Fig. 5:**
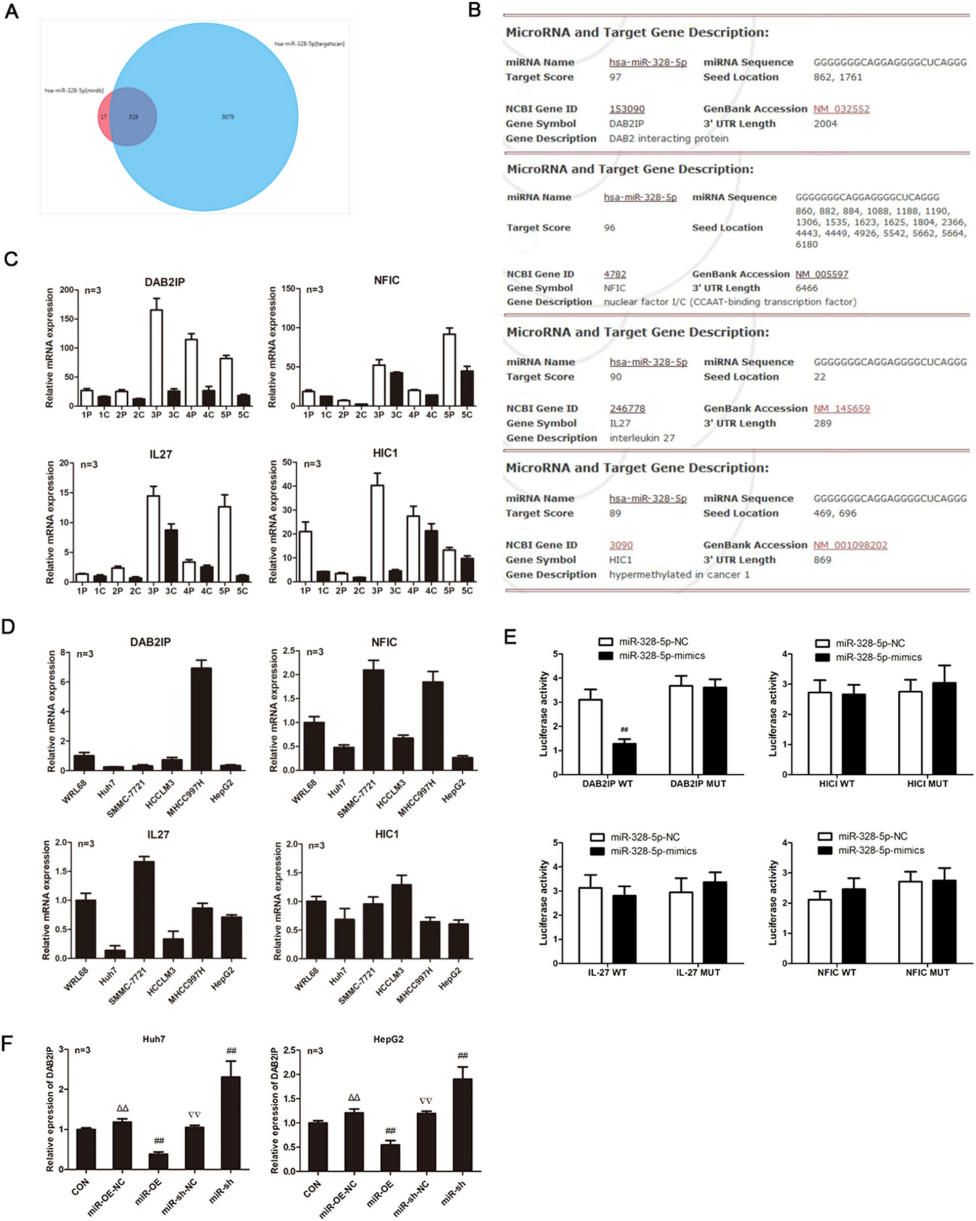
Bioinformatics and validation of the potential targeted mRNAs of miR-328-5p in HCC cells. **a**, **b** Bioinformatics predicted the targeted mRNAs. **c**, **d** Quantitative RT-PCR analysis of the relative levels of the indicated mRNAs in five pairs of HCC tissues and cells. **e** Luciferase assays revealed that transfection with miR-328-5p mimics inhibited the DAB2IP-controlled luciferase activity in HEK293T cells. **f** Altered miR-328-5p expression changed the relative levels of DAB2IP mRNA transcripts in HCC cells. Data are images, or expressed as the mean ± SEM of each group from three separate experiments. ^##^*P* < 0.01 vs. CON; ^▵▵^*P* < 0.01 vs. miR-OE; ^▿▿^*P* < 0.01 vs. miR-sh. OE overexpression, NC transfected with control RNA or lentivirus, Sh transduced with lentivirus for miR-328-5p-specific shRNA.

Next, the potential target genes of miRNA-328-5p were predicted by bioinformatics by using miRDB (http://mirdb.org/) and TargetScanHuman(www.targetscan.org). There were 336 genes predicted as putative target genes of miRNA-328-5p by miRDB, and 339 by TargetScanHuman (Fig. 5a). Because miRNA-328-5p enhanced the malignant behaviors of HCC cells, we searched the putative target genes with tumor-suppressive function. Among the potential target genes of miRNA-328-5p, the *DAB2IP, NFIC, IL-27*, and *HIC1* genes were tumor suppressors (Fig. 5b). Actually, their mRNA transcripts obviously decreased in five HCC specimens, compared with their para-non-tumor liver tissues (Fig. 5c). Their mRNA transcripts also decreased in the majority of HCC cells tested (Fig. 5d). Further luciferase assays revealed that transfection with miRNA-328-5p mimics, but not its mutant, significantly mitigated the DAB2IP-regulated luciferase activity in HEK293T cells (*P* < 0.01, Fig. 5e). However, transfection with either miRNA-328-5p mimics or its mutant failed to alter the HIC1, IL-27, or NFIC-regulated luciferase activity in the same experimental conditions. Finally, while miR-328-5p overexpression significantly decreased DAB2IP mRNA transcripts, miR-328-5p silencing dramatically increased DAB2IP mRNA transcripts in Huh-7 and HepG2 cells (Fig. 5f). Such data suggest that miRNA-328-5p may target the DAB2IP mRNA to enhance the malignant behaviors of HCC.

### DAB2IP attenuates the malignant behaviors of HCC cells

**Fig. 6:**
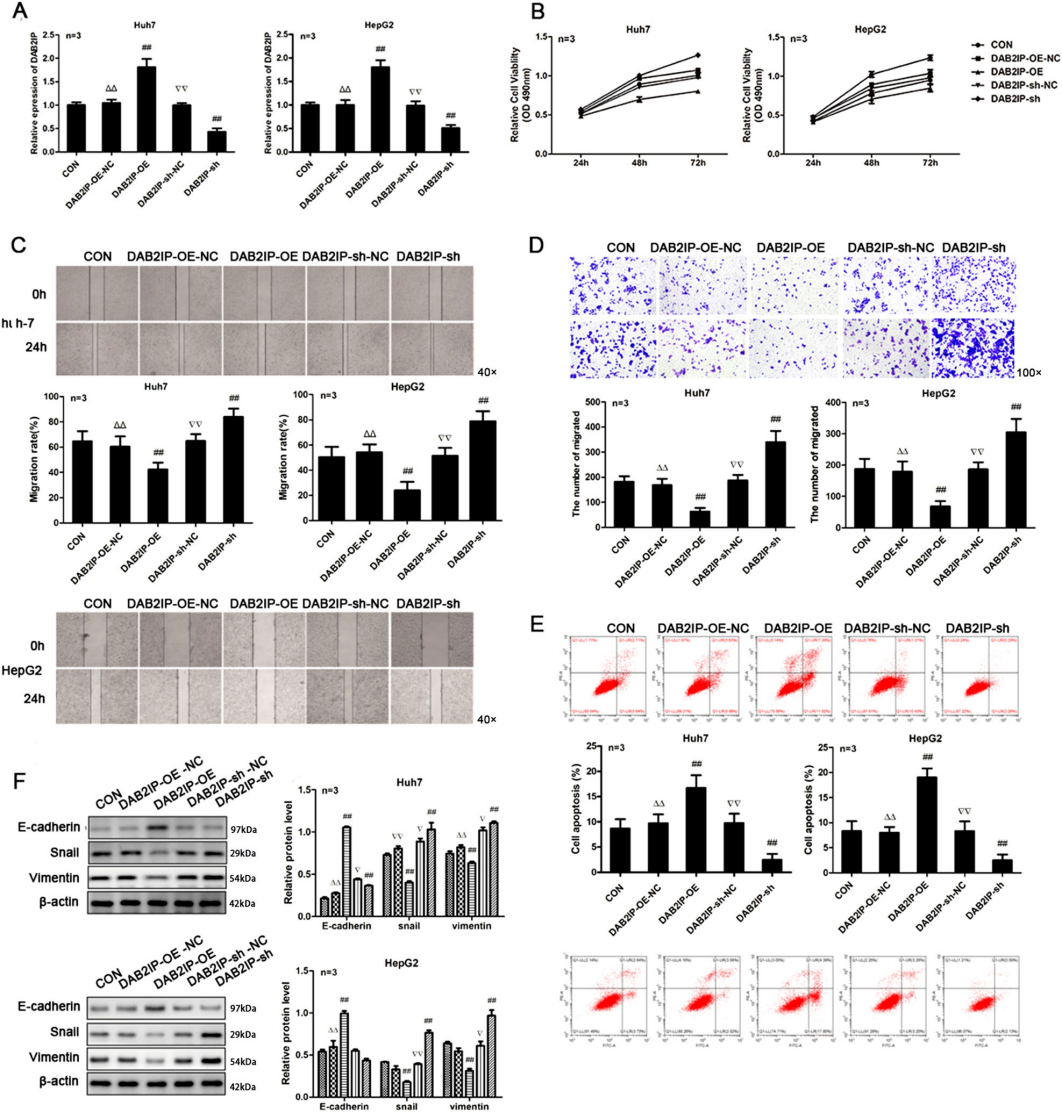
Altered DAB2IP expression changes the malignant behaviors of HCC cells in vitro. Huh-7 and HepG2 cells were transfected with control lentivirus or lentivirus for DAB2IP expression or DAB2IP-specific shRNA expression. **a** The relative levels of DAB2IP mRNA transcripts were determined by quantitative RT-PCR. **b–e** The proliferation, wound healing, invasion, and apoptosis of HCC cells were examined. **f** The relative levels of EMT-relevant protein expression in the different groups of cells were determined by Western blot. Data are images, charts, or expressed as the mean ± SEM of each group from three separate experiments. ^##^*P* < 0.01 vs. CON; ^▵▵^*P* < 0.01 vs. DAB2IP-OE; ^▿▿^*P* < 0.01 vs. DAB2IP-sh. OE overexpression, NC transfected with control RNA or lentivirus, Sh transduced with lentivirus for DAB2IP-specific shRNA.

Previous studies have shown that downregulated DAB2IP expression is associated with poor prognosis of HCC^24,25^. To understand the consequence of miR-328-5p-regulated DAB2IP expression, we generated DAB2IP stably overexpressing and silencing HCC cells (Fig. 6a). Compared with the controls, DAB2IP overexpression significantly inhibited the proliferation, wound healing, and invasion of both Huh-7 and HepG2 cells, and significantly promoted the apoptosis of both Huh-7 and HepG2 cells (*P* < 0.01 for all, Fig. 6b–e). In contrast, DAB2IP silencing displayed opposite effects on these HCC cells. Further, Western blot indicated that DAB2IP overexpression increased the relative levels of E-cadherin, but decreased Snail and Vimentin expression in Huh-7 and HepG2 cells, while DAB2IP silencing exhibited the reverse effects in Huh-7 and HepG2 cells (Fig. 6f). Thus, DAB2IP, like the circRNA-5692, attenuated the malignant behaviors of HCC cells.

### CircRNA-5692 overexpression attenuates the growth of HCC in vivo

**Fig. 7:**
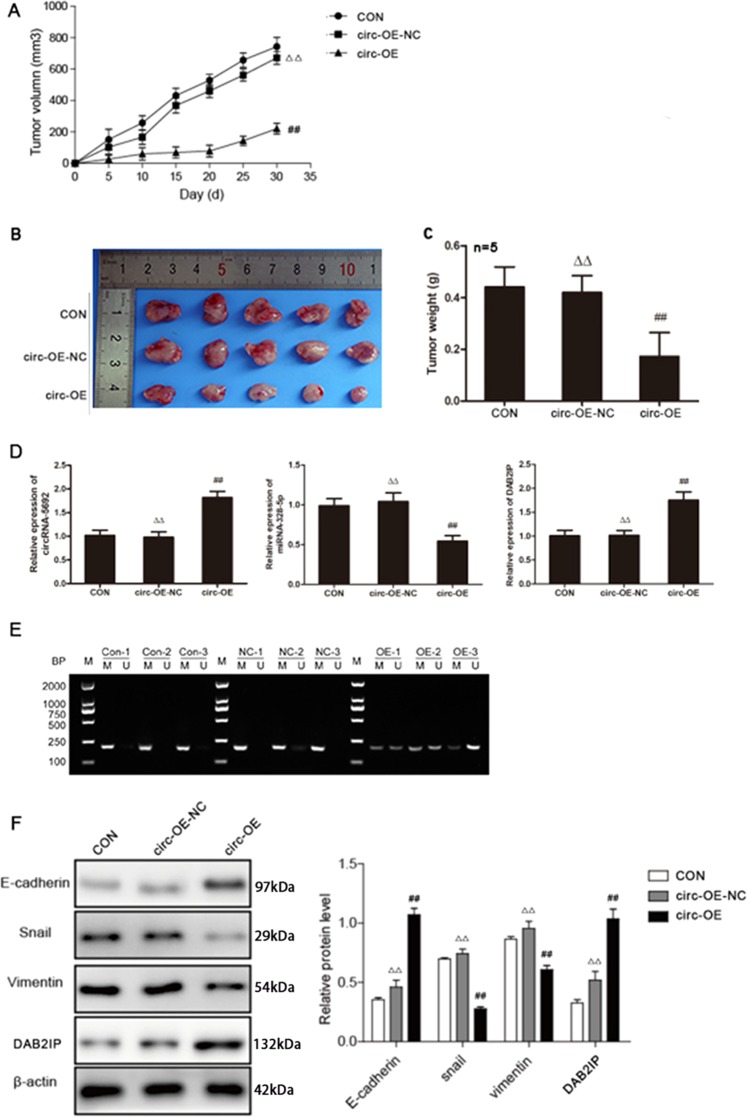
CircRNA-5692 overexpression inhibits the growth of implanted HCC tumors in mice. C57BL/6 nude mice were implanted subcutaneously with Huh-7, Huh-7/NC, or Huh-7/OE cells (n = 3 per group). **a** The dynamic growth of implanted tumors was monitored longitudinally. **b**, **c** The tumor sizes were imaged and their weights were measured. **d** The relative levels of circRNA-5692, miR-328-5p, and DAB2IP mRNA transcripts in tumor tissues were determined by quantitative RT-PCR. **e** The methylation status of the DAB2IP promoter of tumor tissues was determined by PCR. **f** The relative levels of EMT-relevant protein expression in the tumor tissues were determined by Western blot. Data are images, or expressed as the mean ± SEM of each group from three separate experiments. ^##^*P* < 0.01 vs. CON; ^▵▵^*P* < 0.01 vs. circ-OE. OE overexpression, NC transduced with control lentivirus.

Finally, we tested the impact of circRNA-5692 overexpression on the growth of implanted Huh-7 tumors in vivo. C57BL/6 nude mice were injected with control Huh-7 cells, control lentivirus-transduced Huh-7 cells, or circRNA-5692-overexpressing Huh-7 cells to establish solid tumors. Compared with the control mice, circRNA-5692 overexpression significantly attenuated the growth of implanted Huh-7 tumors (*P* < 0.01, Fig. 7a) and reduced the tumor size and weights in mice (Fig. 7b, c). Furthermore, circRNA-5692-overexpressing tumors displayed significantly higher levels of circRNA-5692 and DAB2IP mRNA transcripts, but lower miR-328-5p expression (Fig. 7d). Given that high DAB2IP methylation is commonly observed in various tumor tissues, we evaluated the methylation state of *DAB2IP* in grafted HCC tumors^26^. By using unmethylated PCR primers, we detected *DAB2IP* DNA fragments in the circRNA-5692-overexpressing tumors, but not clearly in the control tumors (Fig. 7e), indicating that the *DAB2IP* methylation was downregulated in the circRNA-5692-overexpressing tumors. Compared with the controls, significantly higher levels of E-cadherin and DAB2IP expression, but lower levels of Snail and Vimentin expression, were detected in the circRNA-5692-overexpressing tumors (Fig. 7f). Therefore, circRNA-5692 overexpression attenuated the growth of implanted Huh-7 tumors in vivo by sponging miR-328-5p to enhance DAB2IP expression.

## Discussion

Tumor cells usually display malignant behaviors, such as rapid proliferation, migration, invasion, and resistance to apoptosis^27^. Previous studies have shown that miR-221 and miR-224 can regulate the proliferation and invasion of HCC cells^28,29^. In this study, we identified differentially expressed circRNAs that were downregulated in clinical HCC tissues, compared with non-tumor liver tissues by microarray. We found that 71 circRNAs were downregulated in HCC tissues, and validated that the randomly selected six circRNAs, particularly for circRNA-5692, decreased their expression in HCC tissues and cells. Further validation in 92 pairs of HCC and non-tumor liver tissues indicated that downregulated circRNA-5692 expression was significantly associated with smaller tumor size, multiple nodules, and higher differentiation of HCC in this population. To the best of our knowledge, this was the first report on downregulated circRNA-5692 expression in HCC, which extended previous reports of other circRNAs^30–33^. The downregulated circRNA-5692 expression suggests that circRNA-5692 may act as a tumor suppressor to attenuate the malignant behaviors of HCC. Actually, we found that circRNA-5692 overexpression attenuated the malignant behaviors of HCC cells in vitro and HCC growth in vivo, accompanied by inhibiting the EMT process. Given that the EMT process is crucial for the invasion and metastasis of HCC^34–36^, the reduced EMT process by circRNA-5692 indicates that circRNA-5692 may be an inhibitor of HCC invasion and metastasis. Hence, circRNA-5692 may be a therapeutic target for inhibition of HCC metastasis, and our findings may shed new light on the pathogenesis of HCC.

It is well known that a circRNA can bind to its targeted miRNAs and act as a ceRNA to sponge these miRNAs and inhibit their activity^19^. While miRNAs bind to the 3′UTR of mRNAs to suppress their translation, and promote their degradation^37^, circRNA through sponging the miRNAs would enhance the miRNA-targeted gene expression. Actually, circRNA Cdr1as can sponge miR-7 to enhance CCNE1 and PIK3CD gene expression and SMMC-7721 cell proliferation^38^. In this study, we identified that circRNA-5692 targeted miR-328-5p in HCC cells because miR-328-5p overexpression mitigated the circRNA-5692-regulated luciferase activity, while circRNA-5692 overexpression decreased miR-328-5p expression in HCC cells, accompanied by modulating the EMT process. More importantly, miR-328-5p overexpression enhanced the malignant behaviors, while its silencing attenuated them in HCC cells. These novel data support the notion that miR-328-5p acts as an oncogenic factor to enhance the malignant behaviors of different types of cancers^39,40^. Therefore, our findings may provide new insights into the molecular regulation of circRNA-569 on the circRNA/miRNA network to regulate the progression of HCC.

We further found that miR-328-5p targeted the *DAB2IP*, one tumor suppressor, because miR-328-5p expression was inversely associated with DAB2IP expression in HCC tissues and cells, and miR-328-5p overexpression mitigated the DAB2IP-regulated luciferase activity. Furthermore, DAB2IP overexpression attenuated the malignant behaviors and inhibited the EMT process of HCC cells, while DAB2IP silencing displayed opposite effects on HCC cells. Given that DAB2IP is a protein in the RAS–GTPase family^26^ and acts as a tumor suppressor for different kinds of tumors^41^, such data indicated that miR-328-5p targeted *DAB2IP* mRNA to attenuate its expression, together with decreased circRNA-5692 expression to reduce its sponging activity, and promoted the progression of HCC. Therefore, the circRNA-5692/miR-328-5p/DAB2IP pathway may be critical for the development and progression of HCC.

In this study, we failed to detect the *DAB2IP* promoter region by PCR by using unmethylated primers in the control HCC tumors, consistent with previous observations that hypermethylation of the *DAB2IP* promoter region is responsible for its downregulated expression in different types of malignant tumors^42,43^. In contrast, we found that circRNA-5692 overexpression decreased the methylation levels of the *DAB2IP* promoter region in the HCC xenograft tumors. We understand that one circRNA or miRNA can target several mRNAs, while one mRNA can be targeted by several miRNAs. Furthermore, circRNAs can directly bind to transcription factors and proteins to regulate their functions. The decreased methylation by circRNA-5692 overexpression may stem from the fact that circRNA-5692 may interact with methyltransferase to decrease the methylation levels of *DAB2IP* promoter region and enhance its expression in HCC. We are interested in further investigating the molecular mechanisms by which circRNA-5692 decreases the methylation of the *DAB2IP* promoter region in the HCC.

In summary, our data indicated that circRNA-5692 was downregulated in HCC tissues and cells, and acted as a tumor suppressor to attenuate the malignant behaviors of HCC cells, accompanied by inhibiting the EMT process. Furthermore, circRNA-5692 effectively sponged miR-328-5p, which targeted the *DAB2IP* to enhance the malignant behaviors of HCC cells, while the DAB2IP effectively suppressed the malignant behaviors of HCC cells. Moreover, circRNA-5692 overexpression attenuated the EMT process and implanted HCC tumor growth in vivo by promoting demethylation in the *DAB2IP* gene. Hence, the circRNA-5692/miR-328-5p/*DAB2IP* pathway may be critical for regulating the development and progression of HCC and may be a therapeutic target for intervention of HCC. Therefore, our findings may shed new light on the pathogenesis of HCC.

## Supplementary information

cddis_author_contribution_form

Reproducibility Checklist
