# Supplementary material for: CircRNA-5692 inhibits the progression of hepatocellular carcinoma by sponging miR-328-5p to enhance DAB2IP expression
Source: Cell Death Dis. 2019 Nov 27;10(12):900. doi: 10.1038/s41419-019-2089-9 (PMC6881381; doi:10.1038/s41419-019-2089-9)
Supplement: Supplementary file 1 — cddis_author_contribution_form [file 41419_2019_2089_MOESM1_ESM.pdf]

DECLARATION OF CONTRIBUTIONS TO ARTICLE

ADMC

Manuscript Number: CDDIS-19-2576R

Journal Name: *Cell Death & Differentiation* (the 'Journal')

Proposed Title of the Contribution:

CircRNA-5692 inhibits the progression of hepatocellular carcinoma by sponging miR-328-5p to enhance DAB2IP expression (the 'Contribution)

Author(s):

Zhenguo Liu, Yaqun Yu, Zebing Huang, Yi Kong, Xingwang Hu, Wei Xiao, Jun Quan, Xuegong Fan (the 'Authors')

For all *CDD* articles, each person named as an author in the published version must be able to show he or she has contributed substantially to the article.

Authorship credit should be based on 1) substantial contributions to conception and design, acquisition of data, or analysis and interpretation of data; 2) drafting the article or revising it critically for important intellectual content; and 3) final approval of the version to be published. Authors should meet conditions 1, 2 and 3.

Any person who cannot be shown to have made a substantial contribution to the article cannot be listed as an author in the final version. The name of any person who is deemed to have made a minor contribution can, however, appear in the Acknowledgments section of the article.

Please complete the table below to indicate the contributions of all named authors to the manuscript.

Author Full Name:	Specification of Contribution to the Manuscript:
Zhenguo Liu	conceived and designed research,collected data and conducted research,analyzed and interpreted data,wrote the initial paper
Yaqun Yu	collected data and conducted research
Zebing Huang	analyzed and interpreted data
Yi Kong	collected data and conducted research
Xingwang Hu	analyzed and interpreted data
Wei Xiao	collected data and conducted research
Jun Quan	conceived and designed research,revised the paper
Xuegong Fan	conceived and designed research,revised the paper

Please complete the table below to indicate the contributions of all named authors to the figures.

Figure 1:

Zhenguo Liu,Jun Quan, Xuegong Fan:conceived and designed research;
Zhenguo Liu, Yaqun Yu, Yi Kong:collected data and conducted research;
Zebing Huang,Xingwang Hu:analyzed and interpreted data.

Figure 2:

Zhenguo Liu, Jun Quan, Xuegong Fan:conceived and designed research;
Zhenguo Liu, Wei Xiao:collected data and conducted research;
Zebing Huang,Xingwang Hu:analyzed and interpreted data.

Figure 3:

Zhenguo Liu, Jun Quan, Xuegong Fan:conceived and designed research;
Zhenguo Liu, Wei Xiao:collected data and conducted research;
Zebing Huang,Xingwang Hu:analyzed and interpreted data.

Figure 4:

Zhenguo Liu, Jun Quan, Xuegong Fan:conceived and designed research;
Zhenguo Liu, Wei Xiao:collected data and conducted research;
Zebing Huang,Xingwang Hu:analyzed and interpreted data.

Figure 5:

Zhenguo Liu, Jun Quan, Xuegong Fan:conceived and designed research;
Zhenguo Liu, Wei Xiao:collected data and conducted research;
Zebing Huang,Xingwang Hu:analyzed and interpreted data.

Figure 6:

Zhenguo Liu, Jun Quan, Xuegong Fan:conceived and designed research;
Zhenguo Liu, Wei Xiao:collected data and conducted research;
Zebing Huang,Xingwang Hu:analyzed and interpreted data.

Signed for and on behalf of the Author(s):	Print Name:	Date:
Zhenguo Liu Quan Jun Xuegong Fan	Zhenguo Liu, Jun Quan, Xuegong Fan	2019/9/25
